# Another "Wart" Story

**Published:** 1900-10-06

**Authors:** 


### ANOTHER "WART" STORY.

Among the curiosities of disease which pathologists must somehow explain and put in line with their other observations before they can dogmatise as to the permanence of organic changes is the disappearance of warts, often apparently under nervous influences of the character of suggestion. As is well known, the stories about warts and their cure by strange devices are infinite, and in many cases are so strange that it is only on the hypothesis of suggestion that they can be explained or even believed. Needless to say, however, the theory that such solid and obvious overgrowths as warty masses can be made to shrivel and die off under the influence of such a mental process as suggestion has bearings which reach far and can hardly be limited to warts alone. A case is related by Dr. Dibble Staple[1] of a girl aged 15 years who had a large number of warts on both her hands. She had counted as many as 94 on the right hand. Having read in one of the medical journals that a number of warts had been cured by vaccination the doctor determined, with the consent of her relatives, to give the plan a trial. He therefore re-vaccinated the patient on June 1st. The vaccination was successful, but no effect was produced on the warts until seven weeks after, when they gradually disappeared, leaving temporary white spots, and when she was seen on August 30th she had no trace of them.

[1] Lancet, September 22nd.

